# Chromosomal Microarray in Children With Developmental Delay: The Experience of a Tertiary Center in Korea

**DOI:** 10.3389/fped.2021.690493

**Published:** 2021-09-15

**Authors:** Eun Hye Yang, Yong Beom Shin, Soo Han Choi, Hye Won Yoo, Hye Young Kim, Min Jung Kwak, Kyung Hee Park, Mi Hye Bae, Ju Hyun Kong, Yun-Jin Lee, Sang Ook Nam, Young Mi Kim

**Affiliations:** ^1^Department of Pediatrics, Pusan National University Hospital, Biomedical Research Institute, School of Medicine, Pusan National University, Busan, South Korea; ^2^Department of Rehabilitation, Pusan National University Hospital, Biomedical Research Institute, School of Medicine, Pusan National University, Busan, South Korea, Busan, South Korea; ^3^Department of Pediatrics, Pusan National University Children's Hospital, Biomedical Research Institute, School of Medicine, Pusan National University, Yangsan, South Korea

**Keywords:** developmental disability, intellectual disability, microarray analysis, pediatrics, autistic disorder

## Abstract

**Background and Objectives:** Chromosomal microarray (CMA) is a first-tier genetic test for children with developmental delay (DD), intellectual disability (ID), autism spectrum disorders (ASDs), and multiple congenital anomalies (MCA). In this study, we report our experiences with the use of CMA in Korean children with unexplained DD/ID.

**Methods:** We performed CMA in a cohort of 308 children with DD/ID between January 2010 and September 2020. We also retrospectively reviewed their medical records. The Affymetrix CytoScan 750 K array with an average resolution of 100 kb was used to perform CMA.

**Results:** Comorbid neurodevelopmental disorders were ASD (37 patients; 12.0%), epilepsy (34 patients; 11.0%), and attention deficit hyperactivity disorders (12 patients; 3.9%). The diagnostic yield was 18.5%. Among the 221 copy number variants (CNVs) identified, 70 CNVs (57 patients; 18.5%) were pathogenic. Deletion CNVs were more common among pathogenic CNVs (PCNVs) than in non-PCNVs (*P* < 0.001). The size difference between PCNVs and non-PCNVs was not significant (*P* = 0.023). The number of included genes within CNV intervals was significantly higher in PCNVs (average 8.6; 0–347) than in non-PCNVs (average 47.5; 1–386) (*P* < 0.001). Short stature and hearing difficulty were also more common in the PCNV group than in the non-PCNV group (*P* = 0.010 and 0.070, respectively).

**Conclusion:** This study provides additional evidence for the usefulness of CMA in genetic testing of children with DD/ID in Korea. The pathogenicity of CNVs correlated with the number of included genes within the CNV interval and deletion type of the CNVs, but not with CNV size.

## Introduction

Developmental delay (DD) is defined as a significant delay—approximately two standard deviations (SD) below the mean—in acquiring early childhood developmental milestone in children under 5 years of age. Global DD is a term often used to describe young children who have a delay in two or more domains of development. The domains include language, gross motor function, fine motor function cognition, social and personal development, and activities of daily living (1). Intellectual disability (ID) is a group of disorders with deficits in adaptive and intellectual functioning, and an age of onset before maturity is reached. Intellectual disability is defined as an intelligence quotient (IQ) score <70 at 5 years of age or older (1). Autism spectrum disorders (ASDs) are characterized by impairments in social communication and social interaction, and restricted and repetitive behaviors. The prevalence of autism is approximately 0.7% worldwide, and has been steadily increasing with changes in diagnostic concepts and criteria (2). Approximately 45% of the patients with autism have ID, with language disorders being the frequent manifestations (2). The prevalence of diverse developmental disabilities, including global DD, ID, and ASD, has been reported to be 15.04% in the United States, and the incidence of DD/ID is approximately 3% in the general population (3, 4).

Chromosomal microarray (CMA) is the first-tier test for the diagnosis of unexplained DD/ID, ASD, and multiple congenital anomalies (MCA) (5). CMA can detect copy number variants (CNVs) under 1 Mb at a much finer resolution than G-banding karyotyping and can identify the size of the rearrangement and the presence of important genes. CMA is also called a cytogenetic microarray, molecular karyotyping, or a genomic copy number array. CMA platforms use array comparative genomic hybridization or single nucleotide polymorphism (SNP) genotyping to identify CNVs, such as gains (microduplications) or losses (microdeletions). It is a difficult challenge for clinicians to interpret the pathogenicity of CNVs. Benign CNVs can be identified not only in the patient but also in the population control group, and the population-based database can help in the interpretation of variants. Pathogenic CNVs are needed to be discriminated from benign CNVs through diverse information about CNVs and databases on known CNVs. The interpretation of variants of unknown significance is a major challenge.

In Korea, CMA in patients with unexplained DD/ID, ASD, and MCA has been covered by the Korean National Health Care Insurance since August 2019. The aim of this study was to confirm the clinical usefulness of CMA in the diagnosis of children with DD/ID in Korea, and to establish clinical characteristics potentially associated with pathogenic CNVs.

## Materials and Methods

### Patients

The clinical records of 315 patients with DD/ID who underwent CMA testing for the diagnosis of DD/ID at the Pusan National University Hospital between January 2010 and September 2020 were reviewed retrospectively. DD was diagnosed when the patient had delayed development in more than one category among five categories (gross motor, fine motor, social and personal, language, and cognitive development) recognized by the Korean Developmental Screening Test for Infants and Children or Bayley Scales of Infant and Toddler Development, Third Edition (6, 7). A diagnosis of global DD was made when the patient exhibited a delay in more than two categories. ID was diagnosed when the patient IQ was <70 on the Korean-Wechsler intelligence scale. ASD diagnostic criteria proposed in the Diagnostic and Statistical Manual of Mental Disorders, 5^th^ edition, were used for ASD assessment. Patients were assessed by pediatricians, physiatrists, and a psychiatrist. The following inclusion criteria were used: (1) individuals diagnosed with DD/ID below 19 years of age, (2) follow-up for a minimum of 6 months, (3) unexplained DD/ID without any identifiable cause, and (4). DD/ID with or without multiple congenital anomalies with no identifiable cause. Exclusion criteria included DD/ID with identifiable and known causes, that is (1) environmental factors, such as child abuse and neglect; (2) prenatal causes, such as congenital infection and congenital malformations; (3) perinatal causes, such as birth asphyxia, hypoxic-ischemic encephalopathy, and prematurity; and (4) other conditions known to be associated with DD/ID, such as thyroid dysfunction, inborn errors of metabolism, neuromuscular disorders, and head trauma.

We retrospectively reviewed medical records and collected data including demographic features, clinical characteristics, and the results of laboratory and neuroimaging tests.

### CMA and Data Analysis

Genomic DNA was extracted from peripheral blood leukocytes. CMA analysis was performed using the Affymetrix Cytoscan® 750k (Affymetrix, Santa Clara, CA, USA) according to the manufacturer's recommendations. This platform includes 550,000 CNV markers and 200,000 SNP markers with an average resolution of 100 kb. The data were visualized and analyzed using the Chromosome Analysis Suite software package (Affymetrix). The February 2009 human reference sequence (GRCh37/Hg19, http://genome.ucsc.edu/) was used as the reference sequence.

All detected CNVs were classified as pathogenic, likely pathogenic, VUS, likely benign, and benign according to the guidelines of the American College of Medical Genetics and Genomics (ACMG) (8, 9). Classification was aided by literature review and the use of public databases, that is, the Database of Genomic Variants (http://dgv.tcag.ca), dvVAR (https://www.ncbi.nlm.nih.gov/dbvar/), DECIPHER (https://decipher.sanger.ac.uk/), UCSC genome browser (https://genome.ucsc.edu/), Online Mendelian Inheritance in Man (OMIM) (https://www.omim.org/), and GeneReviews (https://www.ncbi.nlm.nih.gov/books/NBK1116/).

### Statistical Analysis

Statistical analysis was performed using SPSS, version 27.0 (IBM Corp, Armonk, NY, USA). Descriptive statistics are presented as means and SD. Continuous variables are expressed as means and ranges. Categorical variables are expressed as counts and percentages. Continuous variables were analyzed using independent-samples *t-*test or Mann-Whitney test. Dichotomous variables were analyzed using the chi-square test. Multivariate analysis was used to identify the factors that have contributed to the positive CMA results. Odds ratio (OR) with 95% confidence interval (CI) was used to assess the association between diverse clinical characteristics and pathogenic CNVs. In all analysis, *P*-value < 0.05 was considered statistically significant.

### Standard Protocol Approval

This study was approved by the Institutional Review Boards (IRB) of Pusan National University Hospital (1703-008-006). All studies followed the tenets of the Declaration of Helsinki, and written informed consent was waived from IRB due to the retrospective design.

## Results

### Demographics and Clinical Features

Three hundred and eight patients diagnosed with DD/ID with CMA results were enrolled during the study period. Of the 315 patients who underwent CMA, 7 were excluded for the following reasons: 2 premature infants, 1 fragile X-syndrome, 1 head trauma, 1 congenital hypothyroidism, and 2 abnormal results in tests for the inborn errors of metabolism.

**Figure 1:**
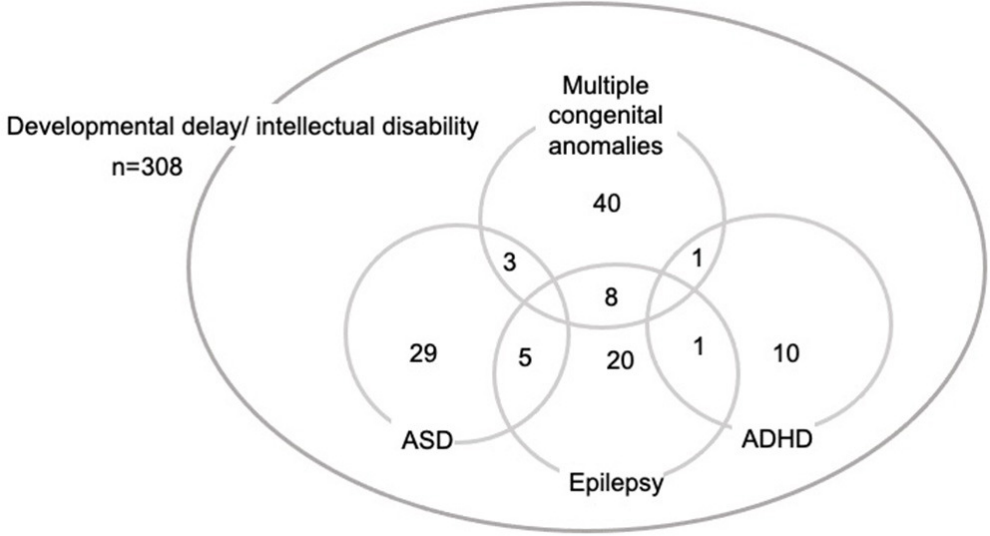
Clinical diagnosis of 308 patients with developmental delay/intellectual disability.

Demographics and clinical characteristics are summarized in Table 1. The age distribution of patients was eight infants (4 weeks−1 year old; 2.6%), 18 toddlers (1–2 years old; 5.8%), 151 preschoolers (2–5 years old: 49.0%), 117 school-aged children (6–13 years: 38.0%), and 14 adolescents (14–19 years: 4.5%). Fifty-two patients (16.9%) had multiple congenital anomalies. In family history, there were 19 patients (6.2%) with DD/ID, 2 patients (0.6%) with ASD, 1 patient with schizophrenia (0.3%). As for abnormal growth, 54 patients (17.5%) had short stature and six patients (1.9%) had tall stature. Forty patients (13%) had microcephaly and 12 patients (3.9%) had macrocephaly. MCA was confirmed in 52 patients (16.9%) and 16 patients (5.2%) had hearing difficulty. Comorbid neurodevelopmental disorders included ASD (37 patients; 12.0%), epilepsy (34 patients; 11.0%), and attention deficit hyperactivity disorders (ADHD; 12 patients; 3.9%). Two or more comorbid disorders were reported in 18 patients (5.8%) (Figure 1). IQ test results were available for 105 patients (34.1%). The levels of ID were profound ID (IQ < 20) (5 patients; 4.8%), severe ID (IQ = 20–35; 12 patients; 11.4%), moderate ID (IQ = 36–49; 23 patients; 21.9%), mild ID (IQ = 50–69; 48 patients; 45.7%), borderline (IQ = 70–80; 12 patients; 11.4%), and normal IQ (>80; 5 patients; 4.8%).

**Table 1:** Demographics and clinical characteristics.

**Characteristics**	**Total (*n* = 308)**
**Demographics**	
**Sex, No. (%)**	
Male	171 (55.5)
Female	137 (44.5)
**Age (years)**	5.93 ± 3.51
Infant (4 week-1year)	8 (2.6)
Toddler (1–2 years)	18 (5.8)
Preschooler (2–5 years)	151 (49.0)
School-aged child (6–13 years)	117 (38.0)
Adolescent (14–19 years)	14 (4.5)
**Clinical features, No. (%)**	
**Family history**	
DD/ID	19 (6.2)
ASD	2 (0.6)
Schizophrenia	1 (0.3)
**Growth**	
Short stature	54 (17.5)
Tall stature	6 (1.9)
**Head circumference**	
Microcephaly	40 (13.0)
Macrocephaly	12 (3.9)
**Dysmorphic face**	95 (30.8)
**Multiple congenital anomalies**	52 (16.9)
**Hypotonia**	90 (29.2)
**Hearing difficulty**	16 (5.2)
**Comorbid neurodevelopmental disorders**	
ASD	37 (10.4)
Epilepsy	34 (9.1)
ADHD	12 (3.2)
ASD + Epilepsy	5 (1.6)
Epilepsy + ADHD	1 (0.3)

*ASD, autism spectrum disorder; ADHD, attention deficit hyperactivity disorder; DD, developmental delay; ID, intellectual disability*.

### Metabolic Screening

To rule out treatable causes of neurodevelopmental disorders, metabolic screening—tandem mass screening (183 patients; 59.4%), urine organic acid (177 patients; 57.5%), serum lactate/pyruvate (183 patients; 59.4%), serum ammonia (196 patients; 63.6%), serum creatine kinase (212 patients; 68.8%), very long chain fatty acids (157 patients; 51.0%), and urine glycosaminoglycans and oligosaccharides (127 patients; 41.2%)—and thyroid function tests (206 patients; 66.9%) were performed. Patients with treatable causes of DD/ID in the metabolic screening tests were excluded.

### CMA Results

CMA results were normal in 160 patients (51.9%). Two hundred and twenty-one CNVs were identified in 148 patients (48.1%). The number of CNVs per patient was 1 CNV in 90 patients (29.2%), 2 CNVs in 46 patients (14.9%), 3 CNVs in 9 patients (2.9%), 4 CNVs in 1 patient (0.3%), and 5 CNVs in 1 patient (0.3%). There were 88 microdeletion CNVs (40.4%) and 130 microduplication CNVs (59.6%). In accordance with the ACMG guidelines (8, 9), the identified 221 CNVs were categorized in the following manner: 85 benign CNVs (39.0%), 35 likely benign CNVs (16.1%), 31 VUS (14.0%), 19 likely pathogenic CNVs (8.6%), and 51 pathogenic variants (22.5%) (Table 2).

**Table 2:** Identified CNVs per patient.

		**No. identified CNV per patient**					
	**Total**	**0 CNV**	**1 CNVs**	**2 CNVs**	**3 CNVs**	**4 CNVs**	**5 CNVs**
Patients No. (%)	308	160	90	46	10	1	1
**CNVs No. (%)**							
Total	221	0	90	92	30	4	5
Benign	85(38.5)	0	43(47.8)	30(32.6)	10(33.3)	2(50.0)	0
Likely benign	35(15.8)	0	20(22.2)	9(9.8)	2(6.7)	0	4 (80.0)
VUS	31(14.0)	0	8(7.8)	14(15.2)	9(30.0)	0	0
Likely pathogenic	19(8.6)	0	4(4.4)	13(14.1)	0	2(50.0)	0
Pathogenic	51(23.1)	0	15(16.7)	26(28.3)	9(30.0)	0	1(20.0)

*CNV, copy number variants; VUS, variant of unknown significance*.

Pathogenic/likely pathogenic CNVs (PCNVs) were clinically considered as positive CMA results. Of the 221 CNVs, there were 70 PCNVs (51 pathogenic CNVs+19 likely pathogenic CNVs) and 151 non-PCNVs (85 benign CNVs + 35 likely benign CNVs + 31 VUS). Deletion CNVs were more common in PCNVs (*n* = 43, 61.4%) than in non-PCNVs (*n* = 47, 31.1%) (*P* < 0.001). The average size of CNVs was longer in non-PCNVs, but there was no significant difference between non-PCNVs (34,771,850.6 bp, range 38,586–606,737,107 bp) and PCNVs (27,309,028.6, range 55,976–1,260,351,215) (*P* = 0.230). The number of included genes from the OMIM database was significantly higher in PCNVs (average 47.5, 1–386) than that in non-PCNVs (average 8.6, 0–347) (*P* < 0.001) (Table 3).

**Table 3:** Cytogenetic features of the identified CNVs (total CNVs = 221).

**Features**	**Non-PCNVs (*n* = 151)**	**PCNVs (*n* = 70)**	***P-*value**
Deletion/duplication (No.)	47/104	43/26	0.000^*^
Size, average (bp, range)	34,771,850.6 (38,586–606737107)	27,309,028.6 (55,976–1,260,351,215)	0.230
OMIM gene No. (range)	8.6 (0–347)	47.5 (1–386)	0.000^*^

*OMIM, online Mendelian inheritance in man; PCNV, pathogenic/likely pathogenic copy number variants*.

* *P < 0.05*.

Seventy PCNVs were identified in 57 patients in total 308 patients (18.5%). Therefore, the diagnostic yield of CMA was 18.5%. We divided the patients into two groups: the positive PCNV group, which consisted of patients with one or more PCNVs (*n* = 57, 18.5%), and the negative PCNV group (*n* = 251, 81.5%). The number of detected CNVs (benign/likely benign/VUS/likely pathogenic/pathogenic) was significantly higher in the positive PCNV group than that in the negative PCNV group (*P* < 0.001). Short stature and hearing difficulty were also more common in the positive PCNV group than in the negative PCNV group (*P* = 0.010 and 0.070, respectively). Some clinical features, including maleness, microcephaly, major anomaly, minor anomaly, and ADHD were more common in the positive PCNV group than in the negative PCNV group, but the differences were not significant (Table 4). IQ was tested in 105 patients and there was no significant difference of the severity of ID between the positive PCNV group and the negative PCNV group.

**Table 4:** Comparison of the clinical characteristics between the group without a PCNS and the group with PCNVs.

**Multivariate analysis parameter**	**without a PCNV (*n* = 251)**	**With PCNVs (*n* = 57)**	**OR**	**95% CI**	***P-*value**
Male	140 (55.8)	31 (54.4)	2.141	0.796–5.754	0.131
Family history of DD/ID	17 (6.8)	3 (5.3)	0.998	0.271–3.675	0.998
Short stature	37 (14.7)	17 (29.8)	2.410	1.231–4.718	0.010^*^
Tall stature	5 (2.0)	1 (1.8)	1.377	0.132–14.337	0.789
Microcephaly	29 (11.6)	11 (19.3)	1.090	0.437–2.720	0.854
Macrocephaly	10 (4.0)	2 (3.5)	1.144	0.213–6.150	0.875
Dysmorphism	73 (29.1)	22 (38.6)	1.319	0.677–2.568	0.416
Major anomaly	33 (13.1)	10 (17.5)	4.839	0.472–49.554	0.184
Minor anomaly	130 (51.8)	36 (63.2)	3.105	1.070–9.009	0.037^*^
MCA	41 (16.3)	11 (19.3)	0.193	0.020–1.856	0.154
Hypotonia	71 (28.3)	19 (33.3)	0.945	0.473–1.889	0.873
Hearing difficulty	10 (4.0)	6 (10.5)	2.630	0.924–7.931	0.070
ASD	31 (12.4)	5 (10.5)	1.319	0.478–3.640	0.593
Epilepsy	29 (11.6)	7 (12.3)	0.947	0.366–2.455	0.911
ADHD	8 (3.2)	3 (5.3)	2.543	0.568–10.591	0.229

*ADHD, attention deficit hyperactivity disorder; ASD, autism spectrum disorders; DD, developmental delay; ID, intellectual disability; MCA, multiple congenital anomalies; PCNV, pathogenic/likely pathogenic copy number variants*.

* *P < 0.05*.

Several known microdeletion and duplication syndromes were detected (Table 5). Among the microdeletion syndromes, steroid sulfatase deficiency and 22q13 deletion syndrome (Phelan-McDermid syndrome) (*n* = 4, respectively) were the most common followed by Wolf-Hirschhorn syndrome (*n* = 3). Cri-du-Chat syndrome, DiGeorge syndrome, and Miller-Dieker syndrome were diagnosed in two patients each. Among the microduplication syndromes, there were 7q11.23 duplication syndrome (*n* = 2), Williams-Beuren syndrome (WBS) (*n* = 2), 16p13.11 microduplication syndrome (*n* = 2), and 22q11 duplication syndrome (*n* = 2). Additionally, uniparental disomy (UPD) was identified in four patients. In three patients with UPD of chromosome 15, Prader-Willi syndrome was identified in two patients and Angelman syndrome was identified in one patient using methylation polymerase chain reaction. One patient had UPD of chromosome 9.

**Table 5:** Detected genetic syndromes and specific genetic aberrations.

**Type**	**Genetic syndromes and genetic aberrations**	**No**.
Gain	7q11.23 duplication syndrome	2
	Williams-Beuren syndrome (WBS)	2
	16p13.11 recurrent microduplication	2
	22q11 duplication syndrome	2
	7p duplication syndrome	1
	15q duplication syndrome	1
	16p11.2 microduplication syndrome	1
	Cat-Eye syndrome (type 1)	1
	17p11.2 duplication syndrome (Potocki-Lupski syndrome)	1
	Xp11.22p11.23 microduplication syndrome	1
	Xq28 duplication syndrome	1
	XYY syndrome	1
Loss	22q13 deletion syndrome (Phelan-Mcdermid syndrome)	4
	Steroid sulfatase deficiency (STS)	4
	Wolf-Hirschhorn syndrome	3
	Cri-du-Chat syndrome	2
	DiGeorge syndrome	2
	Miller-Dieker syndrome	2
	1q21.1 recurrent microdeletion	1
	6p25.3 microdeletion syndrome	1
	8p23.1 deletion syndrome	1
	16p11.2p12.2 microdeletion syndrome	1
	16p13.11 recurrent microdeletion	1
	Angelman syndrome	1
	Xp deletion (mild Turner syndrome)	1
Other	Prader-Willi syndrome	2
	Angelman syndrome	1
	t(10:18) unbalanced translocation	1
	t(10:21) recombinant chromosome	1
	UPD (9)	1

*UPD, uniparental disomy*.

## Discussion

DD/ID can be caused by multiple factors, such as prenatal exposure to drugs, infections, trauma, perinatal hypoxia, postnatal infection, or environmental factors. Even after detailed history taking and careful physical examination, the cause of DD/ID in many patients remains unknown. However, causative genetic reasons can often be identified. Over the past few decades, an increasing number of CNVs have been reported to be associated with DD/ID, and CMA is the first-tier test for the genetic diagnosis of DD/ID, ASD, and MCA (5). In Korea, CMA has been covered by the Korean National Health Care Insurance since September 2019; therefore, CMA has not been actively implemented until recently.

CNVs can be found in healthy persons, and determination of the pathogenicity of CNVs is challenging. Therefore, it is important to retrospectively and periodically reanalyze and reinterpret CNVs. In particular, VUS should be reinterpreted in response to the growing knowledge and data. Patients should be informed of the possibility of reinterpretation and changes in pathogenicity prior to the test.

In general, larger CNVs may contain additional genes and be more significant. Girirajan et al. (10). reported that patients with ID and MCA have larger CNVs than patients with ID alone. However, the pathogenicity of CNV is not determined only by size. Very large CNVs may not be clinically significant, and small CNVs can be pathogenic (11, 12). In this study, the sizes of PCNVs and non-PCNVs were not significantly different (*P* = 0.230), and the average CNV size was larger in non-PCNVs (Table 3). With respect to the types of CNVs, deletion was significantly more common in PCNVs (61.4%) than in non-PCNVs (31.1%) (*P* < 0.001). The number of OMIM genes included in CNVs was significantly higher in PCNVs than that in non-PCNVs (*P* < 0.001). Therefore, the pathogenicity of CNVs might be associated with the number of included OMIM genes and deletion CNVs, not the size of the CNVs.

The diagnostic yields of CMA in DD/ID were reported to be 5.3–35% when it covered the entire genome (5, 13, 14). In this report, the diagnostic yield of CMA was 18.5%, which is similar to that reported in previous studies. The number of CNVs, including benign, likely benign, VUS, likely pathogenic, and pathogenic, was significantly higher in the positive PCNV group than that in the negative PCNV group (*P* < 0.001). The number of CNVs might be associated with the possibility of genetic diagnosis. Dysmorphism has been reported to be associated with the presence of pathogenic CNVs (15). MCA and major anomalies have also been reported to be associated with CNVs (16–18). There are reports that short stature is related to PCNVs, but there are reports that it is not (15, 19). In this study, sex, family history of DD/ID, tall stature, head circumference, ID severity, MCA, hypotonia, and comorbidities were not significantly associated with positive CMA results. Only short stature and hearing difficulty were associated with the presence of PCNVs. However, microcephaly, moderate/severe/profound ID, dysmorphism, major/minor anomaly, MCA, and hypotonia were more common in positive PCNVs. As the correlation between phenotypes and pathogenicity of CNVs was not consistent in this study and in previous studies, it is difficult to predict the pathogenicity of CNVs based on phenotypic clues in unexplained DD/ID.

SNP arrays can identify CNVs and loss of heterozygosity. UPD is the inheritance of both homologous chromosome pairs from a parent. UPD might be of no clinical consequence if it does not contain an autosomal recessive disease or imprinted genes with different expression patterns, depending on the parent origin. An SNP array can detect UPD if a large block of homozygosity is detected in a single chromosome. In this study, we identified four patients with UPD. For the three patients with UPD of chromosome 15, two were confirmed to have Prader–Willi syndrome and one patient was diagnosed with Angelman syndrome through methylation studies. The clinical phenotypes associated with UPD of chromosome 9 are not well-characterized, and imprinted genes are rare on this chromosome. Therefore, the identified UPD of chromosome 9 may not be the reason for DD in this patient.

Additional genetic diagnostic tests were performed in some patients in the negative PCNV group. The tests were selected depending on the patient phenotype. Ten patients were diagnosed based on additional specific genetic tests; congenital myotonic dystrophy with a mutation in *DMPK* (*n* = 2), spinal muscular atrophy type 2 with a mutation in *SMN1* (*n* = 2), Prader-Willi syndrome confirmed by methylation test (*n* = 1), Rett syndrome with a mutation in *MECP2* (*n* = 1), fragile X syndrome with a mutation in *FMR1* (n=1), Sotos syndrome with a mutation in *NSD1* (*n* = 1), Crouzon syndrome with a mutation in *FGFR2* (*n* = 1), and Menkes disease with a mutation in *ATP7A* (*n* = 1). In two patients with Cri-du-Chat syndrome, karyotyping revealed the same results. With detailed history taking and careful physical examination, unnecessary tests can be avoided. Forty-one patients without a positive result after CMA and specific genetic tests were further tested using whole exome sequencing; however, the results are not described in this report. A total of 87 patients had the results of karyotype. Four patients of them had results consistent with the CMA results. There was no balanced translocation.

This study had several potential limitations, such as the retrospective study design, small sample size, limited phenotypic information, and heterogeneous patient groups. The CMA results of the parents were not available for many patients because CMA was unavailable in the clinical setting before September 2019 in Korea. Additionally, the CMA used in this study can diagnose mosaicism of more than 20% of all chromosomes and low-level mosaicism of <20% could not be detected. We believe that future studies should focus on deep phenotyping, using diagnostic techniques for detecting low-level mosaicism in a large number of patients with DD/ID.

In summary, the diagnostic yield of CMA in unexplained DD/ID was 18.5%. This study strongly supports the usefulness of CMA in the clinic as a first-tier genetic diagnostic test for children with unexplained DD/ID in Korea. The pathogenicity of CNVs correlated with a number of included OMIM genes within the CNV interval and deletion type CNVs, but not with the size of the CNVs. Short stature and hearing difficulty correlated with pathogenic CNVs. Given the results of this and previous studies, it is difficult to predict pathogenic CNVs using phenotypic clues.

## Data Availability Statement

The original contributions presented in the study are included in the article/supplementary materials, further inquiries can be directed to the corresponding author/s.

## Ethics Statement

The studies involving human participants were reviewed and approved by the Institutional Review Boards (IRB) of Pusan National University Hospital. Written informed consent to participate in this study was provided by the participants' legal guardian/next of kin.

## Author Contributions

Y-JL, SN, and YK: study conception and design. MB, JK, SC, and HY: data collection. YK, HK, KP, and MK: analysis and interpretation of results. EY and YS: draft manuscript preparation. All authors contributed to the article and approved the submitted version.

## Conflict of Interest

The authors declare that the research was conducted in the absence of any commercial or financial relationships that could be construed as a potential conflict of interest.

## Publisher's Note

All claims expressed in this article are solely those of the authors and do not necessarily represent those of their affiliated organizations, or those of the publisher, the editors and the reviewers. Any product that may be evaluated in this article, or claim that may be made by its manufacturer, is not guaranteed or endorsed by the publisher.
